# Effectiveness of *Bacillus licheniformis*-Fermented Products and Their Derived Antimicrobial Lipopeptides in Controlling Coccidiosis in Broilers

**DOI:** 10.3390/ani11123576

**Published:** 2021-12-16

**Authors:** Yu-Hsiang Yu, Chia-Min Wu, Wei-Jung Chen, Kuo-Feng Hua, Je-Ruei Liu, Yeong-Hsiang Cheng

**Affiliations:** 1 Department of Biotechnology and Animal Science, National Ilan University, Yilan 26047, Taiwan; yuyh@niu.edu.tw (Y.-H.Y.); wjchen@niu.edu.tw (W.-J.C.); kuofenghua@gmail.com (K.-F.H.); 2 Department of Animal Science and Technology, National Taiwan University, Taipei 106, Taiwan; wmandywmandy@gmail.com; 3 Institute of Biotechnology, National Taiwan University, Taipei 106, Taiwan; 4 Agricultural Biotechnology Research Center, Academia Sinica, Taipei 115, Taiwan

**Keywords:** antimicrobial lipopeptide, surfactin, *Bacillus licheniformis*, broiler, coccidiosis

## Abstract

**Simple Summary:**

Coccidiosis is an important health problem in broilers, caused by infection with a highly contagious intestinal parasite of the genus *Eimeria*. Anti-coccidial drugs are widely used for the prevention of coccidiosis in broilers. However, multi-resistance coccidia is a potential threat to poultry production. In this study, we evaluated the potential of *Bacillus licheniformis*-fermented products (BLFP) and their derived antimicrobial lipopeptide, surfactin, on the prevention of coccidiosis in broilers. Results demonstrate that BLFPs and their derived antimicrobial lipopeptide, surfactin, exhibit anti-coccidial activity in vitro and in vivo.

**Abstract:**

This study aimed to investigate the potential of *Bacillus licheniformis*-fermented products (BLFP) and their derived antimicrobial lipopeptide, surfactin, for the prevention of coccidiosis in broilers. Broilers were fed BLFP at 1.25 and 5 g/kg under *Eimeria tenella* challenge. At the end of experiment (35 days), the growth performance, survival rate, cecal morphology, cecal lesion scores, oocyst-count index, and anti-coccidial index were analyzed. The effects of the BLFP-derived surfactin on oocyst sporulation and sporozoite morphology in *Eimeria* species were also investigated in vitro. Results showed that BLFP supplementation at 1.25 and 5 g/kg improved cecal morphology and increased the survival rate of broilers under *E. tenella* challenge. Supplementation with 1.25 g/kg of BLFP reduced the lesion scores in the cecum of *E. tenella*-challenged broilers, while the oocyst-count index was reduced in broilers given 5 g/kg of BLFP. The anti-coccidial index of the 1.25 g/kg of BLFP-treated group was greater than 160, compared with the *E. tenella*-challenge-only group. Furthermore, surfactin inhibited *Eimeria* oocyst sporulation and disrupted sporozoite morphology. These results demonstrate that BLFPs and their derived antimicrobial lipopeptide, surfactin, exhibit anti-coccidial activity in vitro and in vivo. BLFP may be used as a natural feed additive for the prevention of coccidiosis in broilers, and 1.25 g/kg can be considered the optimum dosage.

## 1. Introduction

Coccidiosis is one of the most prevalent enteric diseases in poultry, caused mainly by genus *Eimeria* species. Coccidiosis is estimated to cause up to USD 3 billion in economic loss per year in poultry production due to high mortality and reduced growth performance [1]. Although anti-coccidial drugs are available for the prevention and treatment of coccidiosis, the multiple-drug-resistant strain of *Eimeria* species has become a potential threat to poultry production worldwide [2]. Therefore, exploration of natural alternative strategies for controlling coccidiosis in the poultry industry is an urgent, unmet need.

Probiotics have been used as an alternative to antibiotics in broilers due to their benefits in improving growth, as well as preventing infection [3,4]. *Bacillus licheniformis*, a Gram-positive endospore-forming probiotic strain, was found in the gastrointestinal tract of chickens and exhibits antibacterial activity against pathogens in vitro through the production of antimicrobial lipopeptides [5,6]. Surfactin, an antimicrobial lipopeptide produced from *B. licheniformis*, can kill pathogens by disrupting bacterial cell membranes [5,7,8]. It has been demonstrated that dietary supplementation of *B. licheniformis* alleviates *Clostridium perfringens*-induced necrotic enteritis and improves growth performance in broilers [9,10,11,12]. Furthermore, *B. licheniformis* is able to reduce the negative consequences of enteric diseases in broilers exposed to mixed coccidia infection [13]. However, the precise mechanisms by which *B. licheniformis* confers protection against *Eimeria* species in broilers are still unclear.

Solid-state fermented products produced from *B. licheniformis* not only contain *B. licheniformis* spores but also have *B. licheniformis*-derived antimicrobial lipopeptides [14]. *B. licheniformis*-fermented products (BLFP) have similar benefits as antibiotics in growth performance in broilers [15]. Dietary supplementation of BLFP alleviates necrotic lesions, ameliorates intestinal morphology, and improves growth performance in *C. perfringens*-challenged broilers [16]. BLFP supplementation also modulates the gut microbial community in broilers [15]. Surfactin isolated from BLFP exhibits antibacterial activity against *Brachyspira hyodysenteriae* and *C. perfringens* in vitro [8]. Furthermore, it has been reported that surfactin exhibits anti-parasitic activity against *Plasmodium falciparum* and *Nosema ceranae* [17,18]. However, the effect of BLFP on the alleviation of coccidiosis is still rarely investigated. Whether *B. licheniformis*-derived antimicrobial lipopeptides also exhibit anti-coccidial activity remains to be confirmed.

In the current study, we investigated the effectiveness of BLFPs and their derived antimicrobial lipopeptides in controlling coccidiosis in vivo and in vitro. The results may provide new insights concerning the use of BLFP for the prevention of coccidiosis in the poultry industry.

## 2. Materials and Methods

### 2.1. Preparation of B. licheniformis-Fermented Products

Detailed information about the preparation of BLFP is described in a previous study [15]. Briefly, *B. licheniformis* (ATCC 12713, Food Industry Research and Development Institute, Hsinchu, Taiwan) was inoculated in a wheat-bran-based solid substrate and incubated at 30 °C. After 6 days of fermentation, the solid-state fermented products were baked for 2 days at 50 °C and then crushed into a fine powder using a grinder. *B. licheniformis* spore quantities and surfactin concentrations in fermented products were 5 × 10^9^ CFU/g and 14.3 mg/g, respectively.

### 2.2. Isolation and Characterization of E. tenella Oocysts

*Eimeria* oocysts were collected from the ceca of infected chickens at local commercial farms. *E. tenella* oocysts ~20 μm in diameter were identified by microscopy and interspecies molecular characterization [19,20]. After identification of *Eimeria* species, *E. tenella* oocysts were sporulated with 2% K_2_Cr_2_O_7_ (10^4^ oocysts/mL) at 25 °C for 72 h and propagated in two-week-old chickens not exposed to anti-coccidial drugs. For animal study and oocyst sporulation analysis, *E. tenella* oocysts were collected from fresh feces of broilers artificially infected with *E. tenella* oocysts, followed by sporulation with 2% K_2_Cr_2_O_7_. Protocol for induction of *Eimeria* oocyst sporulation in vitro was based on a previous study [21].

### 2.3. Animal Study

**Figure 1:**
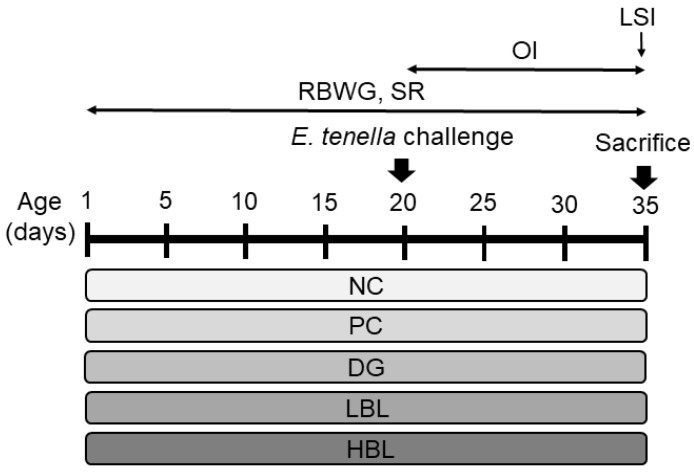
Timeline of the experimental design and sampling during the entire experiment.

Experimental protocols were in accordance with guidelines set by National Ilan University Institutional Animal Care and Use Committee (IACUC, protocol number 107-12). A total of 120 one-day-old healthy male Ross 308 broiler chicks (with an average body weight of 44.4 ± 0.90 g) were obtained from a commercial hatchery. The chicks were allocated to five treatments and six replicates each containing four birds (24 broilers per group) in a completely randomized design. Broilers were reared in stainless-steel cages (89 cm × 56.5 cm × 60 cm). The experimental diets were (1) non-challenged broilers fed a basal diet (NC); (2) *E. tenella*-challenged broilers fed the basal diet (PC); (3) *E. tenella*-challenged broilers fed the basal diet supplemented with 6 mg/kg of the anti-coccidial drug maduramicin (DG); (4) *E. tenella*-challenged broilers fed the basal diet supplemented with 1.25 g/kg of BLFP (LBL); and (5) *E. tenella*-challenged broilers fed the basal diet supplemented with 5 g/kg of BLFP (HBL). The basal diet (Table 1) was formulated to meet or exceed broiler requirements according to the National Research Council [22]. No antibiotics or coccidiostats were included in the diets. Feed and water were provided ad libitum during the 35-day duration of the experiment. The lighting schedule and the room temperature throughout the experiment were provided according to breeder recommendations [23]. Broilers were vaccinated on days 4 and 14 by nose-drop administration with combined Newcastle disease-infectious bronchitis multivalent vaccines (Zoetis, Parsippany, NJ, USA). The birds in the unchallenged group (NC) were orally gavaged with distilled water, whereas birds in challenged groups (PC, DG, LBL, and HBL) were orally inoculated at 20 d of age with 10,000 *E. tenella* oocysts in distilled water. The timeline of the experimental design is shown in Figure 1. Mortality and clinical health status were monitored daily. Body weight, average daily gain, average daily feed intake, and feed-conversion ratio were calculated.

### 2.4. Cecal Morphology

Broilers were humanely slaughtered at the end of the experiment by carbon dioxide inhalation. Five replicates (body weights were close to their group means) were used for cecal morphology evaluation (*n* = 5). The tissue was sectioned at a thickness of 5 μm (3 cross-sections from each sample) using a microtome (Thermo Fisher Scientific, Waltham, MA, USA), mounted on microscope slides, and stained with hematoxylin and eosin. Each section was captured with a digital camera coupled to an Olympus CKX41 microscope (Olympus Corporation, Tokyo, Japan). Measurements of villus height, crypt depth, and villus-height-to-crypt-depth ratio in the cecum of broilers were based on a previous study [24]. Villus height, crypt depth, and villus-height-to-crypt-depth ratio were examined from 10 villi in each bird.

### 2.5. Measurement of Anti-Coccidial Index

The relative body-weight gain (RBWG) between non-challenged broilers (NC) and *E. tenella*-challenged broilers (PC, DG, LBL, and HBL) was calculated from days 1 to 35. The survival rate (SR) of broilers was recorded daily. For lesion-score index (LSI) analysis, three broilers per replicate were humanely slaughtered at the end of the experiment by carbon dioxide inhalation. Both ceca were collected from each bird for macroscopic lesion-score evaluation according to previously described methods [25]. For oocyst-count index (OI), feces from two broilers per replicate were freshly collected daily in a separate cage from days 21 to 35 and pooled. Oocysts per gram of feces was determined via the McMaster’s counting technique, and OI was calculated according to previously described methods [26]. Timeline of sampling during the entire experiment is shown in Figure 1. Anti-coccidial index (ACI) was calculated as follows: ACI = (RBWG (%) + SR (%)) − (LSI) + (OI)). An ACI between 160 and 180 was scored as a marked anti-coccidial effect, between 140 and 159 as moderate, between 120 and 139 as slight, and values below 120 as inactive [27].

### 2.6. E. tenella Oocyst Sporulation Analysis

BLFP-derived surfactin was isolated by acid precipitation method. Briefly, BLFP supernatant was adjusted to pH 2.0 with 6N HCl and incubated at 4 °C for 24 h. The precipitate was harvested by centrifugation at 15,000× *g* rpm for 30 min and then dissolved in distilled water. The mixture was then lyophilized and dissolved in methanol. The surfactin concentration was quantified using high-performance liquid chromatography. Unsporulated *E. tenella* oocysts collected from the ceca of infected chickens were used for oocyst sporulation analysis. *E. tenella* oocyst sporulation was conducted in an aqueous solution of 2% K_2_Cr_2_O_7_ (10^4^ oocysts/mL) with or without 100 μg/mL of BLFP-derived surfactin at 25 °C for 72 h. Sporulated and unsporulated oocysts were counted, and the percentage of sporulation was estimated by microscopic observation by counting the number of sporulated oocysts in a total of 100 oocysts.

### 2.7. Eimeria Species Sporozoite Morphology

In order to avoiding bacterial contamination on the surface of *Eimeria* oocysts and to acquire better images in a scanning electron microscope, a commercial coccidial vaccine (Coccivac-B, Intervet Inc., Omaha, NE, USA) containing live *E. tenella*, *E. acervulina*, and *E. maxima* oocysts was used for *Eimeria* species sporozoite morphology analysis. A total of 10^5^ oocysts were broken by shaking at 6 m/s for 20 min in a 2 mL tube containing zirconia silica beads (BioSpec, Bartlesville, OK, USA), and the released sporocysts were pooled by centrifugation at 1300× *g* for 10 min by adding 1 mL of 50% Percoll (Sigma-Aldrich, St. Louis, MO, USA). The collected sporocysts were incubated in 1 mL of 0.25% trypsin (Thermo Fisher Scientific, Waltham, MA, USA) and 4% taurodeoxycholic acid (Sigma-Aldrich) at 37 °C for 120 min. The excysted sporozoites were suspended in 1 mL of 60% Percoll (Sigma-Aldrich), and the sporozoites were harvested after centrifugation at 1300× *g* for 10 min. Sporozoites (105) were treated with 5, 10, and 20 μg/mL of BLFP-derived surfactin at 37 °C for 60 min. The sporozoites were then fixed with 2.5% glutaraldehyde, washed with 0.1 M potassium phosphate buffer (pH 6.8), dehydrated in a graded ethanol-water series to 95% ethanol (35–95%), and put into a critical point dryer (CPD 030, Bal-Tec AG, Balzers, Liechtenstein). The specimens were coated using a sputter coater (Bal-Tec AG, Balzers, Liechtenstein), then examined using a scanning electron microscope (JSM-6510, JEOL Ltd., Tokyo, Japan).

### 2.8. Statistical Analysis

Replicates were used as the experimental unit. Power analysis was used to calculate the appropriate sample size for this study. Statistical analysis was performed using SAS software (version 9.4, 2012; SAS Institute, Cary, NC, USA). The normally distributed data were subjected to one-way ANOVA analysis, and Tukey’s honestly significant difference test was used for multiple comparisons. Significant differences were determined at *p* < 0.05.

## 3. Results

### 3.1. Effect of BLFP on the Prevention of Coccidiosis in Broilers

No significant differences were observed for body weight, average daily gain, and feed-conversion ratio between groups during the entire experimental period, with the exception of average daily feed intake (Table 2). Average daily feed intake was significantly increased at 22 to 35 days and 1 to 35 days of age in the *E. tenella*-challenge-only group compared with the control group (*p* < 0.05) (Table 2). The anti-coccidial drug maduramicin, in combination with *E. tenella* challenge, increased the average daily feed intake at 22 to 35 days of age compared with the control group (*p* < 0.05) (Table 2).

Supplementation with 1.25 g/kg of BLFP in *E. tenella*-challenged broilers increased cecal villus length compared with those in the control and *E. tenella*-challenge-only groups (*p* < 0.05) (Table 3). Supplementation with BLFP at 1.25 and 5 g/kg in combination with *E. tenella* challenge decreased the crypt depth in the cecum of broilers compared with the control and *E. tenella*-challenge-only groups (*p* < 0.05) (Table 3). The ratio of villus length to crypt depth in the cecum was increased in broilers given BLFP at 1.25 and 5 g/kg in combination with *E. tenella*-challenge groups compared with the other groups (*p* < 0.05) (Table 3).

No significant difference was observed in relative body-weight gain between groups over the whole trial period (day 1 to 35) (Table 4). The survival rate of broilers was reduced in the control group (95.8%), the *E. tenella*-challenge-only group (95.8%), and maduramicin in combination with *E. tenella*-challenge group (91.7%), whereas BLFP at 1.25 and 5 g/kg and in combination with *E. tenella* challenge increased the survival rate of broilers to 100% (Table 4). The lesion scores in the ceca were reduced in the 1.25 g/kg of BLFP in combination with *E. tenella* challenge group compared with the *E. tenella*-challenge-only group (*p* < 0.05) (Table 4). *E. tenella* challenge only increased the oocyst-count index in broilers compared with the control group, whereas 5 g/kg of BLFP in combination with *E. tenella* challenge reduced the oocyst-count index in broilers (*p* < 0.05) (Table 4). The anti-coccidial index of broilers fed only a basal diet, *E. tenella* challenge only, anti-coccidial drug in combination with *E. tenella* challenge, 1.25 g/kg of BLFP in combination with *E. tenella* challenge, or 5 g/kg of BLFP in combination with *E. tenella* challenge were 158.4, 139.1, 157.1, 162.2, and 154.3, respectively (Table 4).

### 3.2. Anti-Parasitic Activity of BLFP-Derived Surfactin on Eimeria Oocysts and Sporozoites

**Figure 2:**
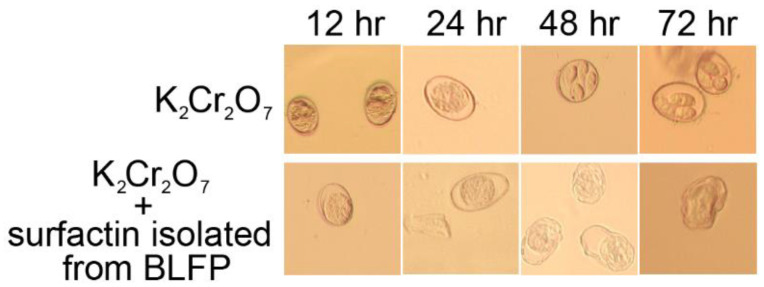
*Bacillus licheniformis*-fermented-product-derived surfactin inhibits *E. tenella* oocyst sporulation *in vitro*. *E. tenella* oocysts were treated with 2% K_2_Cr_2_O_7_ in combination with 100 μg/mL of surfactin at 25 °C for 12, 24, 48, and 72 h. Magnification was 400×. Three experiments (*n* = 3) were carried out, and one representative experiment is shown.

**Figure 3:**
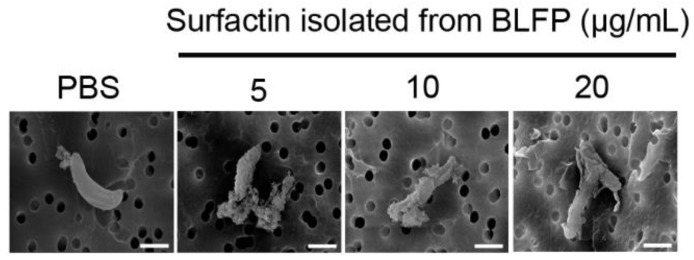
Scanning electron microscope images of *Eimeria* sporozoites illustrating the anti-parasitic activity of *Bacillus licheniformis*-fermented-product (BLFP)-derived surfactin. *Eimeria* sporozoites were treated with 5, 10, and 20 μg/mL of surfactin at 37 °C for 60 min. White bars indicate a length of 2 μm. Three experiments (*n* = 3) were carried out, and one representative experiment is shown.

*E. tenella* oocyst sporulation was increased at 48 and 72 h after stimulation with 2% K_2_Cr_2_O_7_ solution (Table 5 and Figure 2), whereas surfactin isolated from BLFP totally eliminated *E. tenella* oocyst sporulation and disrupted the oocyst morphology (*p* < 0.05) (Table 5 and Figure 2). The results of scanning electron microscopy examinations show that the morphology of *Eimeria* sporozoites was disrupted by BLFP-derived surfactin at 5, 10, and 20 μg/mL (Figure 3).

## 4. Discussion

Coccidiosis caused by *Eimeria* species represents a devastating impact on the poultry industry due to high mortality and significant costs resulting from prophylaxis and treatment. The life cycle of *Eimeria* species begins when sporulated oocysts are picked up and swallowed by chickens. The sporozoites are released from oocysts after grinding in the gizzard and enzymatic digestion in the gut. The sporozoites then imbed in the intestinal lining and develop into merozoites, then multiply several times, damaging tissue [28]. Therefore, controlling the avian coccidiosis is a huge challenge in the poultry industry due to the complexity of life cycle of *Eimeria*. The overuse of drugs for the prevention of coccidiosis in poultry has resulted in the development of multiple drug-resistant strains of *Eimeria* species [2]. Hence, probiotics or probiotic-derived metabolites have been considered as alternative anti-coccidial strategies. It has been reported that *B. licheniformis* supplementation alleviates intestinal-lesion scores, reduces fecal oocyst counts, and improves the body-weight gain in broilers challenged with mixed coccidia infection [13]. Supplementation of *B. licheniformis* can normalize the gut microbiota disorder caused by *C. perfringens* and coccidia-induced necrotic enteritis in broilers [29,30]. Our previous study demonstrated that BLFP had similar benefits to those antibiotics on body weight and daily weight gain in broilers [16]. BLFP can alleviate *C. perfringens*-induced necrotic enteritis in broilers [15]. Furthermore, BLFP supplementation modulates gut-microbiota composition and exhibits anti-coccidial activity in broilers exposed to mixed coccidia infection [26]. In the current study, we further demonstrated that 1.25 g/kg BLFP improved survival rate, reduced cecal-lesion scores, and increased anti-coccidial index in broilers exposed to *E. tenella* challenge. Taken together, these findings demonstrated that BLFP can reduce the effects of *E. tenella*-induced coccidiosis in broilers.

Surfactin is a lipopeptide biosurfactant produced by *B. licheniformis*, and its structure consists of a peptide loop of seven amino acids and a hydrophobic fatty-acid chain [31,32,33]. It has been demonstrated that surfactin is generally ineffective against Gram-negative bacteria but has a broad spectrum of antimicrobial activity against Gram-positive bacteria, such as methicillin-resistant *S. aureus* and *Listeria monocytogenes* [34,35]. Our previous study indicated that surfactin isolated from BLFP can induce death and inhibit growth of *B. hyodysenteriae* and *C. perfringens* in vitro [8]. It has been reported that surfactin exhibits anti-parasitic activity against *P. falciparum* and *N. ceranae* [17,18]. Surfactin can inhibit NAD+ and acetylated peptide formation in *P. falciparum*, thereby impairing intraerythrocytic growth of *P. falciparum* [17]. Surfactin reduces *N. ceranae* parasitosis by direct exposure to *N. ceranae* spores or when ingested by honey bees [18]. In the present study, we further demonstrated that surfactin isolated from BLFP not only inhibited the sporulation of the *Eimeria* oocyst but also disrupted *Eimeria* sporozoite morphology in vitro. However, the anti-parasitic mechanisms of surfactin are not well-characterized. It is proposed that surfactin may insert into lipid bilayer to form membrane pores, solubilize the membrane, and destabilize membrane permeability by channel formation [36]. Thus, the precise mechanism by which surfactin interacts with *Eimeria* oocysts and sporozoites to exert anti-parasitic activity remains to be investigated. Taken together, BLFP-derived surfactin exhibits not only anti-bacterial activity but also anti-coccidial activity in vitro. It has been demonstrated that *Eimeria* sporozoites appear swollen and bulgy after treatment with ionophore anticoccidial for 60 min [37]. In this study, the morphology of *Eimeria* sporozoites was completely disrupted after treatment with 5 μg/mL of antimicrobial lipopeptides for 60 min. Comparison of anti-coccidial efficacy of anticoccidial drugs and *B. licheniformis*-derived antimicrobial lipopeptides in vitro is also needed in the future. Gut microbiota plays a critical role in the control and prevention of coccidiosis. *E. tenella* infection disrupts the integrity of the cecal microbiota and promotes the establishment and growth of potentially pathogenic bacteria, such as *C. perfringens* [38,39,40,41]. Previous studies revealed that probiotic supplementation has the potential to provide protection against coccidiosis in broilers [42,43]. Furthermore, dead probiotics are able to inhibit *E. tenella* sporozoite invasion into Madin-Darby bovine kidney cells, implying steric interference is involved in the anti-coccidial mechanism [43]. *Lactobacillus* species inhibit *E. tenella* invasion by adhering to Madin-Darby bovine kidney cells [44]. Our previous study showed that BLFP supplementation modulates the cecal microbiota of broilers under coccidial challenge by increasing the abundance of the genus *Lactobacillus* [24]. In addition, probiotics administered with a coccidiosis vaccine also enhanced protection against *E. tenella* challenge [42]. These findings indicate that intestinal probiotics (such as *Lactobacillus*, *Enterococcus*, *Bifidobacteria*, and *Pedicoccus*) showed anti-coccidial activity in the gut. Therefore, adhesion of probiotics to the *Eimeria* attachment sites on intestinal epithelial cells and secretion of anti-coccidial metabolites are possible anti-coccidial mechanisms of BLFPs. In the current study, BLFP-derived antimicrobial lipopeptides also exhibited direct anti-coccidial activity by inhibiting *Eimeria* oocyst sporulation and disrupting sporozoite morphology. Taken together, these findings indicating that gut-microbiota modulation and anti-coccidial activity of lipopeptides are involved in the mode of anti-coccidial action of BLFPs.

Intestinal microbiota, gut morphology, and intestinal lesions are closely related to the growth performance of broilers under coccidial challenge [24,38,39,40]. However, the body weight of BLFP-treated broilers under *E. tenella* challenge was not promoted in the present study, although gut morphology and cecal lesions were improved. There is no clear explanation for these results, but we can speculate that the difference in survival rate among groups may be associated with body weight. In the present study, the survival rates of *E. tenella*-challenge-only and anti-coccidial-drug groups were 95.8% and 91.7%, respectively. All dead birds were observed after coccidial challenge and those birds were weak and had low body weight. The birds in BLFP-treated groups survived at the end of experiment and may be recovering from infection after *E. tenella* challenge. However, the body weight of living birds in BLFP-treated groups is comparatively lower than that of living birds in *E. tenella*-challenge-only and anti-coccidial-drug groups (birds with low body weight in these two groups died). In addition, the body weight of the infected broilers (*E. tenella* challenge only) at 35 d of age was greater than that of uninfected broilers (no *E. tenella* challenge) in the present study. Since cecal lesions were observed in uninfected broilers, we think *C. perfringens* (a prevalent pathogen in the environment and gut) may exist in the environment, and all broilers are susceptible to infection by *C. perfringens*. *C. perfringens* infection leads to death (clinical) or growth retardation (sub-clinical) in the control group. Chickens in the *E. tenella*-challenge-only group may have also been infected with *C. perfringens* but died immediately due to *E. tenella* challenge. Therefore, the body weight of living birds in the *E. tenella*-challenge-only group is comparatively heavier than that of living birds in the control group. Anti-coccidial index has been developed as a method to evaluate resistance to anti-coccidial medication by calculation of composite indicators [27,45]. Anti-coccidial index incorporates production (percentage survival and percentage relative weight gain), pathological (lesion score), and parasitological (oocyst count) traits. Animal vaccine studies have widely used anti-coccidial index as a method to evaluate anti-coccidial vaccine efficacy. Although it did not reach statistical significance, the anti-coccidial index of BLFP-treated groups (162.2 of LBL and 154.3 of HBL) was increased in comparison with the *E. tenella*-challenge-only group (139.1) and was almost completely (LBL) or nearly (HBL) back to normal levels (158.4 of uninfected group).

In this study, broilers were reared in cages throughout the whole experiment, and body weight was less than expected at the end of the experiment (Aviagen management handbook), although all diets were formulated to meet or exceed National Research Council recommendations for broilers [21]. According to the Aviagen management handbook, a floor-litter rearing system with advanced environmental control is used to optimize the growth of Ross 308 broilers for commercial purposes. Previous studies have reported that the body weight of broilers from the floor-litter group is greater than that of broilers from the cage group [46,47]. In addition, undefined management stress may also exist in the environment that simultaneously interferes with the growth performance of broilers. For instance, *C. perfringens* may exist in the environment, and broilers are susceptible to infection by *C. perfringens* since cecal lesions are observed in the control group. Therefore, the difference between rearing systems and environmental pathogens is a possible reason for the lower body weight of broilers observed in the present study.

The differences between the current and previous study [25] are: (1) The current study used *E. tenella* oocysts collected from infected chickens for induction of coccidiosis, whereas a commercial coccidial vaccine was used to induce coccidiosis in the previous study. *E. tenella* oocysts collected from infected chickens are closer to the real-world circumstances of coccidial infection. In addition, the anti-coccidial drug was also included for comparison of anti-coccidial activity. (2) *E. tenella* specifically targets the ceca of broilers, thereby causing intestinal microbial disturbance and damaging the intestinal morphology of the ceca. The previous study demonstrates that coccidial challenge can induce dysbiosis of cecal microbiota in broilers, whereas BLFPs normalize the cecal microbiota. Modulation of cecal microbiota by BLFPs is one potential mechanism for prevention of coccidiosis in broilers. Here, we further demonstrated that cecal morphology was improved by BLFP supplementation. (3) We found that *B. licheniformis*-derived antimicrobial lipopeptides could directly inhibit *Eimeria* oocyst sporulation and disrupt sporozoite morphology in vitro. The anti-coccidial index in these two studies was increased after BLFP supplementation. The novelty of the current study is in that the vitro model provides a potential anti-coccidial mechanism. Antimicrobial lipopeptides in BLFPs may directly inhibit *E. tenella* oocyst growth in vivo, thereby preventing coccidiosis in broilers. Whether *B. licheniformis*-derived-antimicrobial-lipopeptide-treated *E. tenella* oocysts still exhibit infectivity in broilers remains to be confirmed in the future.

## 5. Conclusions

BLFP supplementation improved cecal morphology, survival rate, and cecal-lesion scores in broilers exposed to *E. tenella* challenge, and 1.25 g/kg can be considered the optimum dosage. *B. licheniformis*-derived surfactin exhibited anti-coccidial activity by inhibiting the life cycle of *Eimeria* species. The findings provide a theoretical basis for the use of BLFPs as a possible substitute for anti-coccidial drugs in poultry production.

## Figures and Tables

**Table 1:** Composition of basal diets.

Ingredients	1 to 17 d	18 to 35 d
Ingredient, g/kg		
Corn, yellow	470.8	584.3
Soybean meal, 36.7% CP	441.0	337.5
Fish meal	50.0	40.0
CaCO_3_, 38%	20.0	20.0
CaHPO_4_	10.0	10.0
Sodium chloride	4.0	4.0
Choline chloride, 50%	0.2	0.2
DL-Methionine, 99.5%	2.0	2.0
Vitamin premix ^1^	1.0	1.0
Mineral premix ^2^	1.0	1.0
Calculated value, g/kg		
Crude protein	230.0	195.0
Methionine + Cystine	10.0	9.0
Lysine	13.4	12.5
Analyzed total phosphorus	7.4	6.7
Analyzed calcium	13.9	13.1
ME, kcal/kg	3373.8	3353.7

^1^ Vitamin premix provided per kg of diet: 10 mg of nicotine amid, 0.02 mg of cholecalciferol, 0.3 mg of folic acid, 2 mg of pyridoxine HCl, 1.8 mg of all-trans-retinyl acetate, 8 mg of cyanocobalamin, 2.2 mg of menadione, 8.3 mg of alpha-tocopheryl acetate, 160 mg of choline chloride, and 20 mg of D-biotin; ^2^ Mineral premix provided per kg of diet: 60 μg of Se, 200 μg of Co (CoSO_4_), 800 μg of I (KI), 2 mg of Cu (CuSO_4_·5H_2_O), 24 mg of Zn (ZnO), 16 mg of Fe (FeSO_4_·7H_2_O), and 32 mg of Mn (MnSO_4_·H_2_O).

**Table 2:** Effect of *Bacillus licheniformis*-fermented products on growth performance of broilers challenged with *Eimeria tenella*.

Item	NC ^1^	PC	DG	LBL	HBL	SEM	*p*-Value
Body weight (g/bird)							
1 d	44.8	44.2	44.5	44.0	44.5	0.17	0.638
21 d	749.1	779.2	739.4	754.2	757.5	12.03	0.886
35 d	1546.7	1688.3	1686.7	1654.0	1545.0	30.93	0.296
Average daily gain (g/d/bird)							
1–21 d	33.5	35.0	33.1	33.8	34.0	0.57	0.884
22–35 d	57.0	64.9	67.7	64.0	56.3	1.81	0.160
1–35 d	42.9	47.0	46.9	45.9	42.9	0.88	0.293
Average daily feed intake (g/d/bird)							
1–21 d	54.1	53.9	52.4	54.2	53.6	0.29	0.298
22–35 d	74.0 ^b^	104.3 ^a^	102.1 ^a^	88.0 ^ab^	90.3 ^ab^	3.11	0.016
1–35 d	62.1 ^b^	74.2 ^a^	72.3 ^ab^	67.7 ^ab^	68.3 ^ab^	1.24	0.027
Feed-conversion ratio							
1–21 d	1.6	1.6	1.6	1.6	1.6	0.03	0.977
22–35 d	1.3	1.6	1.5	1.4	1.6	0.05	0.127
1–35 d	1.5	1.6	1.6	1.5	1.6	0.03	0.390

^1^ NC = No *E. tenella* challenge; PC = *E. tenella* challenge only; DG = *E. tenella* challenge plus 6 mg/kg of maduramicin; LBL = *E. tenella* challenge plus 1.25 g/kg of BLFP; HBL = *E. tenella* challenge plus 5 g/kg of BLFP; SEM = standard error of mean; ^a,b^ Means (*n* = 6) of a row with no common superscript are significantly different (*p* < 0.05).

**Table 3:** Effect of *Bacillus licheniformis*-fermented products on cecal morphology of broilers challenged with *Eimeria tenella*.

Item	NC ^1^	PC	DG	LBL	HBL	SEM	*p*-Value
Villus length (μm)	317.7 ^b^	288.3 ^b^	402.0 ^ab^	441.3 ^a^	388.2 ^ab^	15.63	0.009
Crypt depth (μm)	111.7 ^a^	112.3 ^a^	87.9 ^ab^	64.0 ^bc^	53.5 ^c^	5.54	<0.001
Villus length: Crypt depth	3.0 ^c^	2.6 ^c^	5.1 ^b^	7.4 ^a^	7.9 ^a^	0.48	<0.001

^1^ NC = No *E. tenella* challenge; PC = E. tenella challenge only; DG = *E. tenella* challenge plus 6 mg/kg of maduramicin; LBL = *E. tenella* challenge plus 1.25 g/kg of BLFP; HBL = *E. tenella* challenge plus 5 g/kg of BLFP; SEM = standard error of mean; ^a–c^ Means (*n* = 5) of a row with no common superscript are significantly different (*p* < 0.05).

**Table 4:** Effect of *Bacillus licheniformis*-fermented products on anti-coccidial index of broilers challenged with *Eimeria tenella*.

Item	NC ^1^	PC	DG	LBL	HBL	SEM	*p*-Value
Relative body-weight gain (%)	97.9 ^2^	110.0	109.1	107.0	100.4	2.26	0.267
Survival rate (%)	95.8	95.8	91.7	100.0	100.0	1.58	0.234
Lesion scores	3.0 ^abc^	3.5 ^a^	2.4 ^bc^	2.2 ^c^	3.3 ^ab^	0.13	<0.001
Oocyst-count index	5.3 ^c^	31.8 ^a^	19.6 ^abc^	23.2 ^ab^	13.6 ^bc^	2.24	<0.001
Anti-coccidial index	158.4	139.1	157.1	162.2	154.3	3.39	0.134

^1^ NC = No *E. tenella* challenge; PC = *E. tenella* challenge only; DG = *E. tenella* challenge plus 6 mg/kg of maduramicin; LBL = *E. tenella* challenge plus 1.25 g/kg of BLFP; HBL = E. tenella challenge plus 5 g/kg of BLFP; SEM = standard error of mean; ^a–c^ Means (*n* = 6) of a row with no common superscript are significantly different (*p* < 0.05).

**Table 5:** Effect of *Bacillus licheniformis*-fermented-product-derived surfactin on *E. tenella* oocyst sporulation in vitro.

	Oocyst Sporulation Rate (%)			
Hour	12	24	48	72
2% K_2_Cr_2_O_7_	0	0	95.6 ^a^	96.7 ^a^
2% K_2_Cr_2_O_7_ + 100 μg/mL surfactin isolated from BLFP	0	0	0 ^b^	0 ^b^
SEM ^1^	0	0	21.40	21.62
*p*-value	-	-	<0.001	<0.001

^1^ SEM = standard error of mean, ^a,b^ Means (*n* = 3) in columns and with no common superscript are significantly different (*p* < 0.05).

## Data Availability

The data presented in this study are available on request from the corresponding author.
